# Retrospective analysis of the efficacy of chemotherapy and molecular targeted therapy for advanced pulmonary pleomorphic carcinoma

**DOI:** 10.1186/s13104-015-1762-z

**Published:** 2015-12-18

**Authors:** Yosuke Tamura, Yutaka Fujiwara, Noboru Yamamoto, Hiroshi Nokihara, Hidehito Horinouchi, Shintaro Kanda, Yasushi Goto, Emi Kubo, Shinsuke Kitahara, Kenjiro Tsuruoka, Koji Tsuta, Yuichiro Ohe

**Affiliations:** Department of Thoracic Oncology, National Cancer Center Hospital, Tsukiji 5-1-1, Chuo-ku, Tokyo, 104-0045 Japan; Division of Internal Medicine, Osaka Medical College Hospital, 2-7 Daigakumachi, Takatsuki, Osaka, 569-8686 Japan; Department of Experimental Therapeutics, Exploratory Oncology Research and Clinical Trial Center, National Cancer Center, Tsukiji 5-1-1, Chuo-ku, Tokyo, 104-0045 Japan; Department of Pathology, National Cancer Center Hospital, Tsukiji 5-1-1, Chuo-ku, Tokyo, 104-0045 Japan

**Keywords:** Pleomorphic carcinoma, Chemotherapy, Gefitinib, EGFR mutations

## Abstract

**Background:**

Pulmonary pleomorphic carcinoma (PPC) follows an aggressive clinical course and outcomes are disappointing. Due to its rarity, however, the clinicopathological and molecular characteristics of this disease remain unclear.

**Methods:**

We retrospectively evaluated the efficacy of chemotherapy and molecular targeted therapy in 16 patients with PPC who received chemotherapy or EGFR-TKI. We also investigated the status of *EGFR* mutation, *KRAS* mutation and ALK expression.

**Results:**

On histologic review of the malignant epithelial component, adenocarcinoma was identified in seven cases (43.8 %), large cell carcinoma in four (25.0 %), and squamous cell carcinoma in two (12.5 %). For the sarcomatoid component, 14 cases (87.5 %) had both spindle cell tumor and giant cell and 2 (12.5 %) had giant cell. Eleven patients received cytotoxic chemotherapy as first-line but did not achieve an objective response, although one patient who received docetaxel as second-line achieved a partial response. We also found that one patient achieved long stable disease of about 9 years without progression after receiving cisplatin and gemcitabine treatment. *EGFR* mutation, *KRAS* mutation and ALK expression were investigated in 14 patients whose tumor specimens were available. *EGFR* mutation was observed in 2 (14.3 %) and *KRAS* mutation in 3 (21.4 %), while no patient was positive for ALK expression. One patient harboring *EGFR* exon 19 deletion was treated with gefitinib after postoperative recurrence and achieved a complete response of about 35 months.

**Conclusions:**

Although advanced PPC showed a poor response to chemotherapy, one patient with *EGFR* mutation achieved an extended complete response. We therefore recommend the evaluation of driver gene alteration such as *EGFR* in the treatment of advanced PPC.

## Background

Pulmonary pleomorphic carcinoma (PPC) is rare, with an incidence of 0.1–0.4 % of all non-small lung cancers (NSCLC) [1–5]. According to the World Health Organization classification report in 2004 [4], pulmonary pleomorphic carcinoma is defined as poorly differentiated adenocarcinoma, squamous cell carcinoma or large cell carcinoma, containing a component of spindle or giant cells with a sarcomatoid tumor component of at least 10 % [4]. Although clinical outcome is stage-dependent, it follows a more aggressive clinical course and has a worse prognosis than other histological types of NSCLC [6–10]. Furthermore, some recent reports have noted that PPC is often refractory to chemotherapy regimens which provide active treatment for NSCLC [8–12]. Due to its rarity, no optimal treatment for PPC has yet been established.

In NSCLC, the discovery that somatic alterations of driver gene, including *epidermal growth factor receptor* (*EGFR*) and *anaplastic large kinase* (*ALK*) gene, are found in a subset of lung adenocarcinomas and are associated with sensitivity to molecular target therapy has provided a rationale for the development of therapies in NSCLC [13–15]. Several reports noted that *EGFR* mutations were recognized in 15–20 % of patients with PPC but that the response to EGFR tyrosine kinase receptor inhibitor (TKI) was weak and transient as a consequence of tumor heterogeneity [8, 10, 11, 16–18].

Here, we retrospectively analyzed the efficacy of chemotherapy and molecular targeted therapy in patients with advanced or metastatic PPC, and characterized their somatic alteration status, particularly for *EGFR* mutation, *K*-*ras* mutation, and ALK immunohistochemistry (IHC).

## Patients and methods

### Patient selection

PPC was diagnosed according to the 2004 World Health Organization classification [4]. Diagnoses were based on light microscopy findings and confirmed by IHC examination. The histological diagnosis was reviewed by one of the authors (K.T.). From January 1998 to April 2010, 65 patients were histologically diagnosed with PPC by surgical resection, transbronchial lung biopsy, or computed tomography (CT) guided needle biopsy at our institution. Of these 65, 13 had received chemotherapy and 3 had received concurrent chemoradiotherapy, giving a total of 16 consecutive patients for final enrollment as subjects of this study. The protocol was approved by the institutional review board of National Cancer Centre Hospital and we reviewed the medical records of all of these patients.

### EGFR mutation, KRAS mutation, and ALK-IHC analysis

Activating EGFR mutations (i.e., exon 19 in-frame deletion and exon 21 L858 R missense mutations) and KRAS mutation in exon 2 (codon 12 and codon 13) were examined in paraffin-embedded tumor specimens by high-resolution melting assay using LCGreen (Idaho Technology) on a LightCycler (Roche Diagnostics), as previously described [19]. These PCR products were denatured at 95 °C for 10 min and cooled to 40 °C to promote the formation of heteroduplexes. The LightCycler capillary was transferred to an HR-1 (Idaho Technology), an high-resolution melting assay instrument, and heated at a transition rate of 0.3 °C/s. Data were acquired and analyzed using the accompanying software (Idaho Technology). After normalization and temperature-adjustment steps, melting curve shapes from 78.5 to 85.5 °C were compared between the tumor samples and control samples. Human Genomic DNA (Roche Diagnostics) was used as the negative control sample with wild-type EGFR. Samples revealing skewed or left-shifted curves as compared with the control samples were judged to have mutations without positive controls.

*ALK* gene fusions were analyzed by immunohistochemistry. Four-micrometer-thick sections were deparaffinized. Heat-induced epitope retrieval was performed with targeted retrieval solution (pH 9) (Dako, Carpinteria, CA). The slides were then incubated with primary antibodies against ALK protein (1:40, 5A4; Abcam, Cambridge, UK) for 30 min at room temperature. Immunoreactions were detected using the EnVision-FLEX and LINKER (Dako). The reactions were visualized with 3,3′-diaminobenzidine, followed by counterstaining with hematoxylin.

To evaluate the genetic heterogeneity of PPC, we also investigated EGFR IHC in two different histological types. For immunohistochemical staining, formalin-fixed, paraffin-embedded tissues were cut into 4-μm-thick sections and deparaffinized, then subject to heat-induced epitope retrieval with Target Retrieval Solution (Dako, Carpinteria, CA, USA). The primary antibody used was a rabbit monoclonal antibody against human EGFR with the DEL (E746-A750del) mutation (1:100, clone 6B6, Cell Signaling Technology, Danvers, MA, USA) and a rabbit monoclonal antibody against human EGFR with the L858R mutation (1:200, clone 43B2, Cell Signaling Technology) [20]. Antibodies were diluted in SignalStain (Cell Signaling Technology) and the slides were incubated with each primary antibody for 1 h at room temperature. Immunoreactions were detected using the EnVision Plus system (Dako) and 3, 3′-diaminobenzidine, followed by counterstaining with hematoxylin.

### Assessment and analysis

Clinical information was obtained from medical records. Clinical disease staging was reassessed according to the latest International Union Against Cancer staging criteria [21]. Response to chemotherapy and survival were assessed retrospectively and classified according to the Response Evaluation Criteria for Solid Tumors, version 1.1 [22]. Progression-free survival (PFS) was defined as the time from the first day of chemotherapy to detection of the earliest signs of disease progression or death from any cause. Overall survival (OS) was defined as the time from the first day of chemotherapy to the last day on which the patient was confirmed to be alive or dead from any cause. Survival was estimated using the Kaplan–Meier method. Fisher’s exact and χ^2^ test was used to examine the association of two categorical variables. Differences with probability values of <0.05 were considered statistically significant. STATA version 12 (StataCorp LP, College Station, TX, USA) was used for all analyses.

## Results

### Patient characteristics

Clinical characteristics of the 16 patients who were finally enrolled are listed in Table 1. Median age was 61 years (range 43–77 years). Ten patients (62.5 %) were male and six (37.5 %) were female. Twelve patients (75.0 %) were ex-smokers and their median pack-years was 45.5 (range 17–124). Ten patients (62.5 %) had relapsed after curative surgery, four (25.0 %) were initially diagnosed with advanced disease at stage IV, and two (12.5 %) were diagnosed with locally advanced disease at stage IIIA. Among the relapsed patients who had undergone curative surgery, none had received adjuvant chemotherapy. Two patients with stage IIIA disease and one who had relapse of mediastinal and supraclavicular lymph node metastases received concurrent chemoradiotherapy. Thirteen patients (81.3 %) received systemic chemotherapy, including cytotoxic agents or molecular targeted agents. Ten patients were diagnosed by the resected primary tumor; of the other six, three were diagnosed by CT guided needle biopsy and three by autopsy.

**Table 1:** Patient characteristics

Characteristic	Number of patients (n = 16)
Sex	
Male	10 (62.5 %)
Female	6 (37.5 %)
Age (years)	
Median (range)	61 (43–77)
ECOG-PS	
0	3 (18.8 %)
1	10 (62.5 %)
2	3 (18.8 %)
Smoking status	
Never smoker	4 (25.0 %)
Ex-smoker	12 (75.0 %)
Pack-years^a^	
Median (range)	45.5 (17–124)
Clinical stage	
IIIA	2 (12.5 %)
IV	4 (25.0 %)
Recurrence	10 (62.5 %)
Diagnostic procedure	
Surgery	10 (62.5 %)
CT-NB	3 (18.8 %)
Autopsy	3 (18.8 %)
Pathological features	
Epithelial component	
Adenocarcinoma	7 (43.8 %)
Squamous cell carcinoma	2 (12.5 %)
Adenosquamous carcinoma	1 (6.3 %)
Large cell carcinoma	4 (25.0 %)
NOS	2 (12.5 %)
Sarcomatoid component	
Spindle cell	0 (0.0 %)
Giant cell	2 (12.5 %)
Spindle cell and giant cell	14 (62.5 %)

*CT-NB* CT-guided needle biopsy, *NOS* not otherwise specified

^a^Ex-smoker (n = 12)

### Pathological features

On histologic review of the malignant epithelial component, adenocarcinoma was identified in seven cases (43.8 %), large cell carcinoma in four (25.0 %), squamous cell carcinoma in two (12.5 %), NSCLC in two (12.5 %), and adenosquamous carcinoma in one (6.3 %). For the sarcomatoid component, two cases (12.5 %) had giant cell tumors. Of the 14 cases (87.5 %) who had both spindle cell tumors and giant cell tumors, the spindle cell tumor was dominant in nine (56.3 %) while the giant cell was dominant in five (31.3 %).

### Molecular profile

*EGFR* mutation, *KRAS* mutation and ALK-IHC were investigated in the 14 patients whose tumor specimens were available (Table 2). *EGFR* mutation was observed in 2 (14.3 %) of these 14 patients. The histological features of the patient harboring *EGFR* exon 19 deletion was adenosquamous carcinoma with spindle cell and giant cell tumor and that of the patient harboring *EGFR* L858R mutation in exon 21 was large cell carcinoma with spindle cell and giant cell tumor. *KRAS* mutation was observed in three patients (21.4 %). Two patients with *EGFR* mutation were never smoker, while all patients with *KRAS* mutation were ex-smokers. No patient was positive for ALK-IHC.

**Table 2:** Association between pathological findings and molecular profile

No.	Sex	Diagnostic procedure	Smoking index	EGFR mutation	KRAS mutation	ALK IHC	Pathological features	
							Epithelial component	Sarcomatoid component
1	M	Surgery	2100	Wild	Mutated	Negative	Adeno	Giant cell > Spindle cell
2	F	Surgery	0	Exon 19 del	Wild	Negative	Adenosquamous	Spindle cell > Giant cell
3	M	Surgery	1200	Wild	Wild	Negative	Adeno	Spindle cell > Giant cell
4	F	Surgery	0	Wild	Wild	Negative	Adeno	Spindle cell > Giant cell
5	M	Surgery	400	Wild	Wild	Negative	Adeno	Spindle cell > Giant cell
6	M	Surgery	1800	Wild	Wild	Negative	Squamous	Spindle cell > Giant cell
7	F	Surgery	600	Wild	Wild	Negative	Adeno	Spindle cell > Giant cell
8	F	Surgery	345	Wild	Mutated	Negative	Large	Giant cell > Spindle cell
9	M	Autopsy	495	NA	NA	NA	NOS	Giant cell > Spindle cell
10	M	Biopsy	2480	Wild	Wild	Negative	NOS	Giant cell
11	M	Autopsy	480	Wild	Wild	Negative	Adeno	Giant cell
12	M	Biopsy	1600	Wild	Mutated	Negative	Squamous	Spindle cell > Giant cell
13	M	Surgery	660	Wild	Wild	Negative	Large	Giant cell > Spindle cell
14	M	Autopsy	1160	Wild	Wild	Negative	Adeno	Giant cell > Spindle cell
15	F	Surgery	0	Exon 21 L858R	Wild	Negative	Large	Spindle cell > Giant cell
16	F	Biopsy	0	NA	NA	NA	Large	Spindle cell > Giant cell

*IHC* immunohistochemistry, *NOS* not otherwise specified, *Adeno* adenocarcinoma, *Squamous* squamous cell carcinoma, *Large* large cell carcinoma, *Adenosquamous* adenosquamous carcinoma

**Fig. 1:**
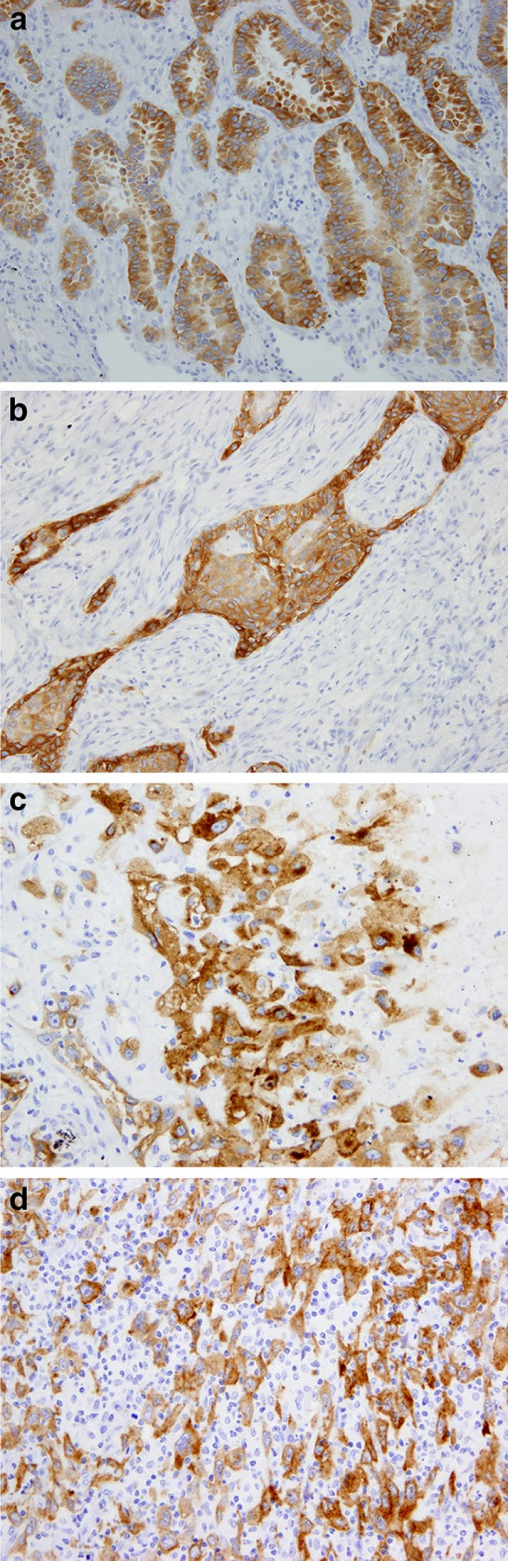
Two representative cases harboring *EGFR* mutation by mutation-specific antibody against *EGFR* mutations in both epithelial and sarcomatoid components. **a** adenocarcinoma component in patient No. 2, ×20; **b** squamous cell carcinoma component in patient No. 2, ×20; **c** sarcomatoid component in patient No. 2, ×20; **d** sarcomatoid component in patient No. 15, ×20

Next, regarding the two cases harboring *EGFR* mutation, we evaluated the mutated EGFR protein expression using a mutation-specific antibody against *EGFR* mutations in both the epithelial and sarcomatoid components. In patient No. 2, we found that IHCs by mutation-specific antibody were positive in adenocarcinoma (Fig. 1a), squamous cell carcinoma (Fig. 1b), and sarcomatoid components (Fig. 1c). And patient No. 15 progressed after concurrent chemoradiotherapy and so we could not check the mutated EGFR protein expression in both the epithelial and sarcomatoid components.

### Treatment and efficacy

**Fig. 2:**
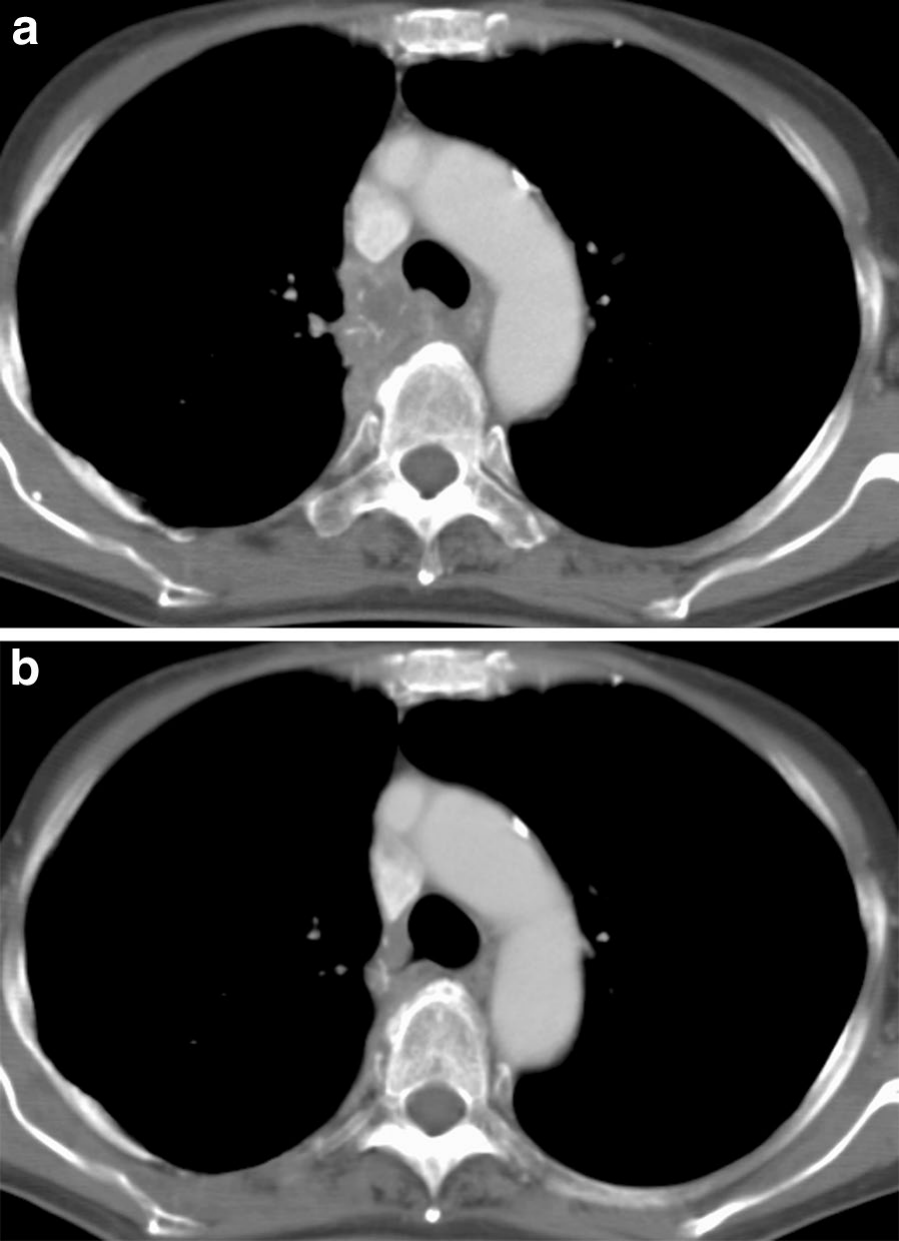
Chest computed tomography images of mediastinal lymph nodes (#4R) before (**a**) and 6 months after gefitinib treatment (**b**)

Of the 13 patients with advanced stage disease, 11 patients received cytotoxic chemotherapy as first-line chemotherapy. Among them, however, no patient achieved an objective response, except one patient (No. 6) who received docetaxel as second-line chemotherapy and achieved a partial response. In all patients, median PFS for first-line chemotherapy was 1.5 (95 % CI 0.6–2.5) months and OS was 7.2 (95 % CI 1.4–10.3) months (Table 3). We also identified one patient (No. 13) who achieved stable disease of about 9 years’ duration without progression after undergoing small intestinal resection for ileus due to a postoperative recurrence lesion in the small intestine and then receiving four cycles of cisplatin and gemcitabine treatment for hilar lymph node and bone metastases. One patient (No. 2) harboring *EGFR* exon 19 deletion was treated with gefitinib after postoperative recurrence of mediastinal lymph node and achieved a complete response of about 35 months (Fig. 2). Two months after she declined further treatment with gefitinib because of hemoptysis, she developed progressive disease in a mediastinal lymph node and thoracic spine metastases. All three patients who received concurrent chemoradiotherapy with cisplatin plus vinorelbine achieved a partial response (Table 3). Although two patients had early recurrence, the third is alive without progression more than 5 years after completing chemoradiotherapy. One patient (No. 15) harboring *EGFR* exon 21 point mutation developed multiple brain metastases and carcinomatous meningitis immediately after chemoradiotherapy and was then treated with best supportive care without EGFR-TKI because of poor general condition.

**Table 3:** Treatment results by systemic chemotherapy and chemoradiotherapy

No.	Sex	Age	Stage	PS	EGFR mutation status	Initial treatment	OR	PFS (mos)	OS (mos)	Outcome
1	M	55	Relapsed	1	Wild	DTX	PD	0.7	7.3	Died
2	F	69	Relapsed	1	Exon 19 del	Gefitinib	CR	35.1	53.9	Died
3	M	60	Relapsed	1	Wild	CBDCA + PTX	PD	0.9	3.7	Died
4	F	45	Relapsed	0	Wild	Gefitinib	PD	1.7	10.7	Died
5	M	63	Relapsed	1	Wild	CBDCA + PTX	PD	0.6	12.7	Died
6	M	62	Relapsed	1	Wild	CDDP + GEM	PD	2.5	18.7	Died
7	F	53	Relapsed	2	Wild	CBDCA + PTX	PD	0.4	1	Died
8	F	55	Relapsed	1	Wild	DTX	PD	0.7	1.4	Died
9	M	56	Advanced	1	NA	CBDCA + PTX	PD	0.6	1.0	Died
10	M	77	Advanced	2	Wild	CBDCA + ETP	PD	1.5	7.2	Died
11	M	43	Advanced	1	Wild	CBDCA + PTX	SD	4.5	10.3	Died
12	M	62	Advanced	2	Wild	CBDCA + PTX	NE	0.4	0.4	Died
13	M	56	Relapsed	0	Wild	CDDP + GEM	NE	109.2	109.2	Alive
14	M	65	Stage IIIA	1	Wild	CDDP + VNR + TRT	PR	5.0	6.6	Died
15	F	66	Relapsed	1	Exon 21 L858R	CDDP + VNR + TRT	PR	3.9	6.9	Died
16	F	66	Stage IIIA	0	NA	CDDP + VNR + TRT	PR	65.4	65.4	Alive

*PS* performance status, *OR* overall response, *PFS* progression-free survival, *OS* overall survival, *DTX* docetaxel, *CBDCA* carboplatin, *PTX* paclitaxel, *CDDP* cisplatin, *GEM* gemcitabine, *ETP* etoposide, *VNR* vinorelbine, *TRT* thoracic radiotherapy, *SD* stable disease, *CR* complete response, *PD* progressive disease, *NE* not evaluable, *PR* partial response

## Discussion

Previous retrospective studies and case reports suggested that PPC is often refractory to the chemotherapy regimens used in active treatment for NSCLC [8–11]. This retrospective study also found that PPC is an aggressive tumor associated with high cell proliferation and that palliative chemotherapy is associated with a poor response in advanced PPC, with a few notable exceptions.

With regard to chemoradiotherapy for locally advanced PPC, all three patients achieved a partial response in our study. Although two patients had early recurrence, the third is alive without progression more than 5 years after completing chemoradiotherapy. Some reports revealed that PPC treated with chemoradiotherapy achieved an objective response without recurrence [10, 11]. These results if confirmed suggest that chemoradiotherapy commonly used for NSCLC should be administered in unresectable stage III disease with pleomorphic carcinoma.

Several recent reports have described cases of PPC that responded to cytotoxic chemotherapy containing platinum and gemcitabine [8–12, 23]. Tamiya et al. mentioned that high expression of human equilibrative nucleoside transporter 1 (hENT1), the major transporter of gemcitabine, might be associated with high sensitivity to gemcitabine in PPC [12]. We also experienced one patient who achieved long-term stable disease without progression after receiving cisplatin and gemcitabine treatment. We propose that cisplatin and gemcitabine treatment may be an active chemotherapy for PPC.

In this study, we also investigated *EGFR* mutation, *KRAS* mutation and ALK-IHC in the 14 patients with PPC whose tumor specimens were available. We found that two (14.3 %) of these patients harbored *EGFR* mutation. One patient treated with gefitinib achieved a complete response, which was moreover sustained for about 35 months. Previous reports suggested that the incidence of *EGFR* mutation was about 15–20 % and that the response to gefitinib was weak and transient [8, 10, 11, 16–18]. Kaira et al. independently investigated *EGFR* mutations in both the adenocarcinomatous and sarcomatoid components of three patients with *EGFR* mutation and detected *EGFR* mutations in the adenocarcinomatous component but not in the sarcomatoid component in all cases. Ushiki et al. revealed a case of PPC whose adenocarcinoma cells had an exon 19 deletion and whose sarcomatous cells had both the deletion 19 and 20 T790 M *EGFR* mutations [16]. In our present study, we could not clearly separate epithelial tumor cells from sarcomatoid tumor cells because tumor tissue consisted of a mixture of both components. We then investigated IHC by mutated EGFR-specific antibody in the two different histological types to evaluate the genetic heterogeneity of PPC. We found that mutated EGFR protein expression was positive in both components. We consider that our results are unlikely to be false positives, because mutation-specific antibodies against EGFR mutation have high specificity in spite of low sensitivity [20]. Tumorigenesis in lung cancer is known to be a multistage process by which monoclonal cancer cells gradually become heterogeneous owing to clonal evolution and genetic/epigenetic instability [24–27]. Applying computational analysis to the deep sequencing data of NSCLC samples, Govindan et al. suggested that EGFR mutation might be acquired at the very initial phase of tumorigenesis [28]. We consider that the difference in the *EGFR* mutational heterogeneity of PPC between the epithelial and sarcomatoid components represents the point at which clonal evolution occurred. Additionally, our results also suggest that driver gene alteration such as *EGFR* should be investigated in the treatment of advanced PPC.

One limitation of our study is that the sample size was small, due to the rarity of this condition. Additionally, 10 of 16 patients diagnosed from the resected primary tumor did not undergone rebiopsy at the start of chemotherapy. For this reason, we did not attempt to identify which component of PPC relapsed.

## Conclusions

All the patients who received concurrent chemoradiotherapy achieved at least a partial response and if confirmed, chemoradiotherapy should be considered an effective modality in locally advanced disease. Although this study demonstrated that advanced PPC responds poorly to chemotherapy, further clinical trials are needed to investigate the role of palliative chemotherapy for PPC, including platinum and gemcitabine treatment. Additionally, one patient with *EGFR* mutation achieved a long-term complete response. We therefore recommend evaluating driver gene alteration, such as *EGFR,* in the treatment of advanced PPC.

## Abbreviations

PPC: pulmonary pleomorphic carcinoma
NSCLC: non-small cell lung cancer
EGFR: epidermal growth factor receptor
ALK: anaplastic large kinase
TKI: tyrosine kinase receptor inhibitor
IHC: immunohistochemistry
CT: computed tomography
PFS: progression free survival
OS: overall survival
